# Effect of residue and weed management practices on weed flora, yield, energetics, carbon footprint, economics and soil quality of zero tillage wheat

**DOI:** 10.1038/s41598-023-45488-3

**Published:** 2023-11-07

**Authors:** R. Puniya, B. R. Bazaya, Anil Kumar, B. C. Sharma, Nesar Ahmed Nesar, R. S. Bochalya, M. C. Dwivedi, Neetu Sharma, Rakesh Kumar, Jyoti Sharma, Ashu Sharma, Swati Mehta

**Affiliations:** 1 AICRP Weed Management, Sher-e-Kashmir University of Agricultural Sciences & Technology, Jammu, 180009 India; 2 College of Agriculture, Kumher (SKNAU, Jobner), Bharatpur, Rajasthan 321201 India; 3 FSR, Sher-e-Kashmir University of Agricultural Sciences & Technology, Jammu, 180009 India; 4 Division of Agronomy, FoA, Sher-e-Kashmir University of Agricultural Science & Technology, Jammu, 180009 India; 5 Shoolini University of Biotechnology and Management Science, Solan, Himachal Pradesh India; 6 KVK, Kathua, Sher-e-Kashmir University of Agricultural Sciences & Technology, Jammu, 18009 India

**Keywords:** Plant sciences, Plant development, Plant stress responses

## Abstract

A two-year field study was conducted during *Rabi* 2018–2019 and 2019–20 to find out the influence of different residue and weed management practices on weed dynamics, growth, yield, energetics, carbon footprint, economics and soil properties in zero-tilled sown wheat at Research Farm, AICRP-Weed management, SKUAST-Jammu. The experiment with four rice residue management practices and four weed management practices was conducted in a Strip-Plot Design and replicated thrice. The results showed that residue retention treatments recorded lower weed density, biomass and higher wheat growth, yield attributes and yields of wheat as compared to no residue treatment. The magnitude of increase in wheat grain yield was 17.55, 16.98 and 7.41% when treated with 125% recommended dose of nitrogen + residue + waste decomposer (RDN + R + WD), 125% RDN + R, and 100% RDN + R, respectively, compared to no residue treatment. Further, all three herbicidal treatments decreased weed density and biomass than weedy treatments. Consequently, a reduction of 29.30, 28.00, and 25.70% in grain yield were observed in control as compared to sulfosulfuron + carfentrazone, clodinafop-propargyl + metasulfuron, and clodinafop-propargyl + metribuzin, respectively. Moreover, 125% RDN + R + WD obtained significantly higher energy output (137860 MJ ha^−1^) and carbon output (4522 kg CE/ha), but 100% RDN had significantly higher net energy (101802 MJ ha^−1^), energy use efficiency (7.66), energy productivity (0.23 kg MJ^−1^), energy profitability (6.66 kg MJ^−1^), carbon efficiency (7.66), and less carbon footprint (7.66) as compared to other treatments. Despite this, treatments with 125% RDN + R + WD and 125% RDN + R provided 17.58 and 16.96% higher gross returns, and 24.45% and 23.17% net outcomes, respectively, than that of control. However, compared to the control, sulfosulfuron + carfentrazone showed considerably higher energy output (140492 MJ ha^−1^), net energy (104778 MJ ha^−1^), energy usage efficiency (4.70), energy productivity (0.14 kg MJ^−1^), energy profitability (3.70 kg MJ^−1^), carbon output (4624 kg CE ha^−1^), carbon efficiency (4.71), and lower carbon footprint (0.27). Furthermore, sulfosulfuron + carfentrazone, clodinafop-propargyl + metasulfuron, and clodinafop-propargyl + metribuzin recorded 29.29% and 38.42%, 27.99%, and 36.91%, 25.69% and 34.32% higher gross returns and net returns over control treatment, respectively. All three herbicides showed higher gross returns, net returns, and benefit cost ratio over control. The soil nutrient status was not significantly affected either by residue or weed management practices. Therefore, based on present study it can be concluded that rice residue retention with 25% additional nitrogen and weed management by clodinafop-propargyl + metasulfuron herbicide found suitable for zero tillage wheat.

## Introduction

Wheat *(Triticum aestivum* L.) is one of the most important cereal crops for the majority of world's population. It contributed about 760 million tonnes to the global food grain basket in the year 2020–2021, from an area of about 219.70 million hectares. World-wide India is firmly occupying the second position among the wheat producing countries after China, while in India, this crop occupies about 30.79 million hectares area and accounts for production of about 107.59 million tonnes with productivity of 3494 kg ha^−1^^1^. Weeds are usually a threat to wheat growth and productivity due to the competition for nutrients, water, light, and space. However, heavy infestation of weeds is one of the foremost constraints to the sustainable production under zero-tillage sown wheat. If do not interfere with weeds growth, it may reduce wheat yield by 15 to 40% depending upon the magnitude, nature, and duration of weed^2^. Farmers have recently been burning significant amounts of crop residues that were left in the field after mechanized harvesting, which interfere with tillage and following operations for the future crop and result in the loss of nutrients and soil organic matter (SOM). Retaining residue on the field improves soil health, soil water conservation, soil productivity, and the environment. However, incorporating residue presents a number of difficulties, including labor-intensiveness, fallow periods, and N immobilization. Therefore, after complete removal of all loose residue from the field, wheat was sown with a zero-tilled seed cum fertilizer drill on sanding rice residue (30–35 cm height) to guarantee timely sowing, avoid extensive tillage operation, and make the best use of available resources under the current scenario.

Adopting no or reduced tillage is a potential alternative for mitigating the difficulties caused by intense tillage in rice and delayed wheat sowing. Zero tillage alleviates the problem of delayed planting while also reducing weeds such as *Phalaris minor* in wheat. The transition from an intense tillage system to a reduced/no-tillage system results in significant changes in weed dynamics, pesticide effectiveness, and weed seed recruitment^3,4^. The present weed control enormously focuses on chemical weed measures. Thus, extensive use of herbicides quickly leads to herbicide resistance in weeds, which is a serious environmental issue and is a matter of concern. Therefore, it’s time to modify weed control approaches to manage the population below the competition threshold level to prevent the problem of herbicide resistance in weeds and increase wheat production in zero-till systems. Agronomic intervention in arable fields can influence weed flora, weed community species composition, and soil seed bank^5^. Consequently, crop rotation, fertilization, tillage, and residue retention are effective measures for controlling weeds. Further, weed management varies amongst different tillage systems, with small seed species favored by conservation tillage systems for the composition of weed community changes, thus requiring different weed control strategies^6^. In addition, weed germination is generally reduced in conservation tillage systems over conventional tillage systems due to less disturbance of soil^7^. As a result, if weeds can be well managed in the early years of conservation tillage, the weed seed bank will be reduced, and ultimately, the potential for high infestation of weeds derived from seeds will decrease^8^.

However, zero tillage can advance the sowing time as the crop can be sown without any field preparation through a single tractor operation using a specially designed seed-cum-fertilizer drill. Therefore, zero tillage is divisible and flexible in operation, allowing farmers to gain more than in a driving situation. Tillage affects weed infestation. Hence, interactions between tillage and weed management measures are widespread in crop production. Thus, zero tillage prevents weed emergence and creates a more suitable atmosphere for early crop establishment^9^. Tillage strongly influences the number and diversity of weed seed banks and has an overriding influence on weed shift.

On other hand, crop residue management is another effective agronomic strategy for weed control because it reduces the amount of sunlight entering the soil surface and provides a safe harbor for bacteria, fungus, insects, and other predators^10^. Further, residue retention on the surface or as mulch proves to result in lower weed density and biomass in broad-leaved as well as narrow-leaved weeds. Additionally, long-term field studies involving the use of rice residue as a component of integrated weed management strategies need to be conducted in the rice–wheat cropping system. Because, rice residue has the potential to sustainably manage the weeds at a low cost. Therefore, the combined adoption of multiple weed control options, both chemical and nonchemical practices such as residue management (retention or incorporation), can help in the effective management of weeds in wheat. Various rice residue management strategies have a variable effect on weed dynamics in wheat due to either physical hindrance or allelopathic interactions. Therefore, the main objective of present study was studying the impact of nitrogen dose, rice residue and chimerical weed management options for controlling weed infestation, energetics, carbon footprint, and economic in zero tillage wheat.

## Materials and methods

### Site description

The present experiment was carried out during *Rabi* seasons of 2018–2019 and 2019–2020 in Research Farm of All India Cordinated Research Project on Weed Management, SKUAST-Jammu, Chatha located in the Shiwalik foothills of the North-Western Himalayas, situated at 32° 40' N latitude and 74° 58'E longitude with an altitude of 332 m above mean sea level. The meteorological data with respect to rainfall, temperature and relative humidity were recorded from the meteorological observatory located very close to the experimental area which reveals that the experimental site was mainly sub-tropical in nature endowed with hot and dry summers followed by hot and humid monsoon seasons. The mean annual rainfall of the location varied from 1050–1115 mm of which about 75 per cent was received from June to September. The soil of the experimental field was sandy clay loam in texture, slightly alkaline in reaction, low in organic carbon and available nitrogen but medium in phosphorus and potassium.

### Experimental treatments and design

Four rice residue management practices namely 100% recommended dose of nitrogen (RDN) + rice residue (R), 125% RDN + R, 125% RDN + R + waste decomposer (WD) and 100% RDN without residue (R) and four weed management practices namely sulfosulfuron + carfentrazone (25 + 20 g/ha), clodinafop-propargyl + metsulfuron (60 + 4 g/ha), clodinafop-propargyl + metribuzin (54 + 120 g/ha) and control were established in a Strip-Plot Design replicated thrice. The dimension of gross and net plot was 6 m × 4.4 m and 5 m × 3.4 m, respectively.

### Crop management

In standing rice plots, direct seeded rice was harvested with combine harvester at a particular height of 30–35 cm above ground and loose residue was completely removed from the field. However, in no residue plots, both loose and standing residue was removed completely from the field. Wheat variety “HD 3086” was used for the study. The seeds were sown with zero till seed cum drill on 17th November 2018 and 16th November 2019. The amount of seed rate was 125 kg ha^−1^. The crop was irrigated twice during 1st and 2nd year the experiment due to evenly distribution of rainfall. The crop was fertilized with 50: 25 kg of P_2_O_5_ and K_2_O ha^−1^, respectively. While, nitrogen was applied as per treatments (100% RDN means 100 kg N/ha). Full doses of phosphorus and potassium along with one-third of nitrogen were applied as basal doses at the time of sowing. The remaining half quantity of nitrogen was applied in two equal splits—at crown root initiation stage and just before ear initiation stage.

### Waste decomposer preparation and use in the field

Organic waste decomposer (WD) solution was prepared and spayed on standing rice residue in the field. For preparation of waste decomposer for 1 ha area used material was 100 ml decomposer, 1 kg besan (chickpea flour), 2.5 kg Jaggery and 200 letter waters. We added 1 kg besan and 2.5 kg jaggery in 200 letter water and mixed it properly and after that added 100 ml of decomposer and kept for 15 days to get ready for use in the field. After 15 days waste decomposer was applied on standing rice residue after sowing of wheat at the rate of 500 letter ha^−1^ as per technical programme.

### Application of herbicide

As per technical programme which mentioned in above section all herbicidal treatments were applied at 30–35 DAS using Knapsack sprayer fitted with flat fan nozzles with 500 letter water ha^−1^. Control plot was maintained herbicide free during both the experimental years.

### Crop studies

The information on the parameters of wheat growth, including plant height (cm), the number of tillers per square meter, and dry matter, was collected 60 days after sowing (DAS) and at harvest. The number of spikes per square meter of the wheat crop was measured before harvest. The average of ten spikes was used to calculate the number of grains per spike. From the net plot area, 1000 seeds were counted and their weight was determined and expressed as 1000-grain weight. Harvested net plot area was left in the field to completely dry before being bundled, with each bundle's weight being recorded. After threshing, wheat grain weight was subtracted from biological yield to produce straw yield.

### Weed studies

The standard quadrant approach developed by Mishra and Mishra^11^ was applied in order to get an accurate reading of the weed density in wheat. The weed density was recorded from a quadrant (0.5 × 0.5 m) chosen at random in each plot at two points at 30 day after sowing (DAS) and harvest and the results were represented as no. m^−2^. Because of the large variation on weed densities that existed among the treatment prior to statistical analysis, the data were transformed using the square root operator (√x + 1). In a similar manner, for the purpose of determining the dry biomass of weeds, the weeds that were collected from an area of 0.25 square metres in wheat were first dried in the sun for two to three days, and then they were oven dried at a temperature of 70 °C until their weight remained constant. Weed dry matter at harvest and was expressed as g m^−2^. The weed control efficiency was worked out based on Mishra and Mishra^11^ equation which is given below:

$$Weed \, \;control \, \;efficiency \, = \frac{Wdc - \, Wdt}{{Wdc}}$$

Where, Wdc = Weed dry weight in control plot (weedy check); Wdt = Weed dry weight in treated plot (treatment).

### Energy budgeting

Both operational (direct) and non-operational (indirect) forms of energy are included in the inputs for the energy. In contrast, non-operational energy included things like seed, manure, and chemical fertilisers and pesticides. Operational energy included things such manual labour, fuel, and machinery and etc. The computation of energy consumption on the basis of energy was carried out using the primary data on a variety of inputs as well as management techniques. The quantity of energy that can be extracted from the product (grain and straw) was determined by multiplying the amount of production with the energy equivalent that corresponds to it. The energy use indices were calculated as per the procedure given by Devasenapathy et al.^12^ and Mittal and Dhawan^13^.

$$Net \, \;energy \, = \, Energy \, \;output \, \left( {MJ \, ha^{ - 1} } \right) \, - \, Energy\; \, input \, \left( {MJ \, ha^{ - 1} } \right)$$

$$Energy \, \;use \, \;efficiency \, = \, Energy \, \;output \, \left( {MJ \, ha^{ - 1} } \right) \, / \, Energy\; \, input \, \left( {MJ \, ha^{ - 1} } \right)$$

$$Energy\; \, productivity \, = \, Economic\; \, yield \, \left( {kg \, ha^{ - 1} } \right) \, /Energy\; \, input \, \left( {MJ \, ha^{ - 1} } \right)$$

$$Energy \, \;profitability \, = \, Net \, \;energy\; \, return \, \left( {MJ \, ha^{ - 1} } \right) \, / \, Energy\;input \, \left( {MJ \, ha^{ - 1} } \right)$$

### Carbon budgeting

Carbon equivalent (CE) was estimated by multiplying the input (diesel, chemical fertilizer and pesticides, water, residue etc.) with its corresponding emission coefficient as given by Lal^14^ and West and Marland^15^. As individual pesticide and herbicide emission coefficients are not readily available, it was anticipated that emissions from manufacturing, shipping, warehousing, and spraying would be consistent within a given pesticide class. The total amount of carbon used and released during crop production was determined by adding up the carbon equivalent of all inputs and products.

$$Carbon \, \;output \, (kg \, CE \, ha^{ - 1} ) \, = \, Total \, \;biomass \, \left( {Economic \, \;yield \, + \, Byproduct \, \;yield} \right) \, \times \, 0.44*$$

*Plant biomass contains on an average 44% carbon content as given by Lal^14^.

$$Carbon\; \, efficiency \, = \, Carbon\; \, output \, / \, Carbon \, \;input$$

The carbon footprint of crop production was calculated as per the methodologies given by Ma et al^16^.

$$\begin{gathered} Carbon \, \;footpr{\text{int}} \, (kg \, CE \, kg^{ - 1} grain) \hfill \\ \;\;\;\; \, = \, Total \, \;carbon\; \, emission\; \, or \, \;input \, (kg \, CE \, ha^{ - 1} ) \, / \, Wheat \, \;yield \, (kg \, ha^{ - 1} ). \hfill \\ \end{gathered}$$

### Statistical analysis

The effect of residue and weed management on weed density, biomass, wheat growth parameters, yield attributes, yields, energetics and carbon footprint were analysed. Analysis of variance of the above-mentioned parameters were performed in OPSTAT software and LSD test with 95% probability level (*P* < 0.05) were used for comparison of treatments. All the weed data were square root transformed prior to analysis to normalize the residuals.

### Ethical approval

Experiment was conducted after taking proper approval from the Sher-e-Kashmir University of Agricultural Sciences & Technology, Jammu and AICRP on Weed Management, ICAR-DWR, Jabalpur. Guidelines of the Sher-e-Kashmir University of Agricultural Sciences & Technology, Jammu were followed for taking data of crop/weed/plants. The seeds of rice and wheat were taken from Sher-e-Kashmir University of Agricultural Sciences & Technology, Jammu.

## Results

### Effect of residue and weed management practices on weed parameters

The major weed flora consisted of *P minor*, *Avena spp*, *Ranunculus arvensis*, *Medicago spp*, *Rumex spp* and *Anagallis arvensis* were observed in the experimental site. The data presented in Tables 1, 2 and 3 exposed that total weed density and biomass was significantly affected by residue and weed management practices. At 30 DAS, residue management practices brought significant variations on total weed density. However, at harvest, 125% RDN + R + WD was found statistically at par with 125% RDN + R and 100% RDN + R, but recorded considerably less grassy, broad-leaved and total weed density as compared to 100% RDN without residue. Among herbicidal weed management practices, significantly less grassy weed density at harvest was noticed in clodinafop-propargyl + metribuzin, which was statistically at par with clodinafop-propargyl + metsulfuron but significantly lower than sulfosulfuron + carfentrazone and control treatments. Whereas, sulfosulfuron + carfentrazone observed significantly less broad-leaved and total weed density at harvest over other treatments. Further, clodinafop-propargyl + metsulfuron and clodinafop-propargyl + metribuzin recorded significantly lower broad-leaved and total weed density over control, but were statistically at par with each other.

**Table 1:** Effect of nitrogen, residue and weed management practices on weed density at 30 DAS of wheat (Average of 2018–2019 & 2019–2020).

Treatments	Grassy weed (No. m^−2^)		Broad-leaved weeds (No. m^−2^)					Total (No. m^−2^)
	*P. minor*	*Avena spp*	*Ranunculus arvensis*	*Medicago spp.*	*Rumex spp.*	*Anagallis arvensis*	Other	
Nitrogen and residue management								
100% RDN* + Rice residue	4.47 (18.96)	2.24 (4.00)	3.98 (14.88)	3.45 (10.88)	2.65 (6.00)	1.94 (2.75)	2.58 (5.67)	(63.14) 8.01
125% RDN + Rice residue	4.39 (18.25)	2.31 (4.33)	3.89 (14.13)	3.39 (10.46)	2.53 (5.42)	1.86 (2.46)	2.58 (5.67)	(60.72) 7.86
125% RDN + Rice residue + Waste decomposer (WD)	4.43 (18.58)	2.25 (4.04)	3.92 (14.33)	3.35 (10.25)	2.56 (5.54)	1.89 (2.58)	2.58 (5.67)	(60.99) 7.87
100% RDN	4.60 (20.13)	2.41 (4.79)	4.37 (18.13)	3.48 (11.13)	2.78 (6.71)	2.04 (3.17)	2.68 (6.21)	(70.27) 8.44
SEm ±	0.08	0.04	0.05	0.04	0.04	0.04	0.06	0.08
LSD (*p* = 0.05)	NS	NS	0.16	NS	0.14	NS	NS	0.29
Weed management								
Sulfosulfuron + carfentrazone (25 + 20 g/ha) at 30–35 DAS	4.43 (18.63)	2.28 (4.21)	4.03 (15.25)	3.39 (10.50)	2.59 (5.71)	1.90 (2.63)	2.58 (5.67)	(62.60) 7.97
Clodinafop-propargyl + metsulfuron (60 + 4 g/ha) at 30–35 DAS	4.41 (18.46)	2.25 (4.08)	4.07 (15.54)	3.33 (10.08)	2.65 (6.04)	1.96 (2.83)	2.61 (5.79)	(62.82) 7.99
Clodinafop-propargyl + metribuzin (54 + 120 g/ha) at 30–35 DAS	4.48 (19.04)	2.27 (4.17)	3.99 (14.96)	3.35 (10.25)	2.62 (5.88)	1.90 (2.63)	2.61 (5.79)	(62.72) 7.98
Control	4.56 (19.79)	2.39 (4.71)	4.09 (15.71)	3.59 (11.88)	2.65 (6.04)	1.97 (2.88)	2.64 (5.96)	(66.97) 8.24
SEm ±	0.04	0.05	0.04	0.07	0.04	0.04	0.05	0.07
LSD (*p* = 0.05)	NS	NS	NS	NS	NS	NS	NS	NS
Interaction	NS	NS	NS	NS	NS	NS	NS	NS

Treatment means were compared at *P* ≤ 0.05 level using least significant difference (LSD); Data in parentheses are original values.

**Table 2:** Effect of nitrogen, residue and weed management practices on weed density at harvest of wheat (Average of 2018–2019 & 2019–2020).

Treatments	Grassy weed (No. m^−2^)		Broad-leaved weeds (No. m^−2^)					Total (No. m^−2^)
	*P. minor*	*Avena spp*	*Ranunculus arvensis*	*Medicago spp.*	*Rumex spp.*	*Anagallis arvensis*	Other	
Nitrogen and residue management								
100% RDN* + Rice residue	2.49 (5.21)	1.76 (2.11)	1.91 (2.67)	2.37 (4.63)	2.00 (3.00)	2.02 (3.08)	2.04 (3.17)	5.45 (28.71)
125% RDN + Rice residue	2.41 (4.79)	1.75 (2.05)	1.80 (2.25)	2.22 (3.92)	1.89 (2.58)	1.91 (2.67)	1.90 (2.63)	5.08 (24.83)
125% RDN + Rice residue + Waste decomposer (WD)	2.36 (4.58)	1.71 (1.93)	1.79 (2.21)	2.23 (3.96)	1.87 (2.50)	1.91 (2.67)	1.94 (2.75)	5.05 (24.50)
100% RDN	2.59 (5.71)	1.84 (2.39)	2.10 (3.42)	2.67 (6.13)	2.22 (3.92)	2.22 (3.92)	2.24 (4.00)	5.99 (34.92)
SEm ±	0.03	0.02	0.05	0.03	0.03	0.04	0.06	0.03
LSD (*p* = 0.05)	0.11	0.06	0.16	0.11	0.12	0.15	0.21	0.12
Weed management								
Sulfosulfuron + carfentrazone (25 + 20 g/ha) at 30–35 DAS	1.99 (2.96)	1.67 (1.79)	2.15 3.63	1.55 (1.42)	1.44 (1.08)	1.14 (0.29)	1.59 (1.54)	3.72 (12.88)
Clodinafop-propargyl + metsulfuron(60 + 4 g/ha) at 30–35 DAS	1.87 (2.50)	1.64 (1.70)	2.29 4.25	1.77 (2.13)	1.67 (1.79)	1.43 (1.04)	1.68 (1.83)	4.05 (15.38)
Clodinafop-propargyl + metribuzin (54 + 120 g/ha) at 30–35 DAS	1.77 (2.13)	1.59 (1.52)	2.34 4.46	1.84 (2.38)	1.77 (2.13)	1.55 (1.42)	1.77 (2.13)	4.14 (16.17)
Control	3.70 (12.71)	2.11 (3.47)	3.99 14.92	3.70 (12.71)	2.83 (7.00)	3.25 (9.58)	2.84 (7.04)	8.34 (68.54)
SEm ±	0.06	0.02	0.03	0.05	0.03	0.03	0.05	0.04
LSD (*p* = 0.05)	0.20	0.08	0.09	0.16	0.10	0.10	0.16	0.15
Interaction	NS	NS	NS	NS	NS	NS	NS	NS

Treatment means were compared at *P* ≤ 0.05 level using least significant difference (LSD); Data in parentheses are original values.

**Table 3:** Effect of nitrogen, residue and weed management practices on weed biomass in wheat (Average of 2018–2019 & 2019–2020).

Treatments	Weed biomass at harvest (g m^−2^)			WCE at harvest
	Grassy weed	Broad-leaved weeds	Total	
Nitrogen and residue management				
100% RDN* + Rice residue	7.03 (48.44)	6.04 (35.47)	9.21 (83.91)	57.23
125% RDN + Rice residue	6.29 (38.58)	5.73 (31.88)	8.45 (70.46)	58.98
125% RDN + Rice residue + Waste decomposer (WD)	6.28 (38.44)	5.53 (29.61)	8.31 (68.05)	59.48
100% RDN	8.12 (64.95)	6.65 (43.19)	10.45 (108.14)	51.72
SEm ±	0.09	0.07	0.11	–
LSD (*p* = 0.05)	0.32	0.26	0.36	–
Weed management				
Sulfosulfuron + carfentrazone (25 + 20 g/ha) at 30–35 DAS	5.06 (24.57)	4.57 (19.88)	6.74 (44.44)	77.37
Clodinafop-propargyl + metsulfuron(60 + 4 g/ha) at 30–35 DAS	4.92 (23.19)	4.98 (23.81)	6.93 (47.00)	75.48
Clodinafop-propargyl + metribuzin (54 + 120 g/ha) at 30–35 DAS	4.85 (22.56	5.18 (25.81)	7.03 (48.37)	74.55
Control	11.00 (120.09)	8.46 (70.66)	13.85 (190.75)	0
SEm ±	0.09	0.11	0.13	
LSD (*p* = 0.05)	0.31	0.37	0.45	
Interaction	NS	NS	NS	

Treatment means were compared at *P* ≤ 0.05 level using least significant difference (LSD); Data in parentheses are original values.

The information on grassy, broad-leaved and total weed biomass (g m^−2^) are given in Table 3 clarified that 125% RDN + R + WD observed lowest grassy, broad-leaved and total weed biomass at harvest which was statistically at par with 125% RDN + R and 100% RDN + R but was significantly lower as compared to 100% RDN. Among weed management practices, at harvest, clodinafop-propargyl + metasulfuron recorded lowest grassy, broad-leaved and total weed biomass which was statistically at par with clodinafop-propargyl + metribuzin and sulfosulfuron + carfentrazone but was significantly lower as compared to control treatments.

### Effect of residue and weed management on growth, yield attributes and yield of wheat

The data depicted in Tables 4 and 5 indicated that residue and weed management practices caused significant variation on wheat growth, yield attributes and yields. Among residue management, 125% RDN + R + WD recorded higher growth parameters namely plant height, numbers of tillers and dry matter accumulation which was statistically at par with 125% RDN + R but significantly higher than that of 100% RDN + R and 100% RDN. While, among weed management practices, at 60 DAS and harvest, all the herbicidal treatments recorded significantly higher growth parameters over control but were statistically at par with each other.

**Table 4:** Effect of nitrogen, residue and weed management practices on wheat growth parameters (Average of 2018–2019 & 2019–2020).

Treatment	Plant height (cm)		Tillers m^−2^		Dry matter (g m^−2^)	
	60 DAS	At harvest	60 DAS	At harvest	60 DAS	At harvest
Nitrogen and residue management						
100% RDN* + Rice residue	41.15	117.24	425.54	418.33	218.86	854.47
125% RDN + Rice residue	42.32	125.13	468.83	462.21	245.46	902.37
125% RDN + Rice residue + Waste decomposer (WD)	42.37	126.26	473.04	465.00	248.00	905.83
100% RDN	35.71	113.15	416.04	412.25	203.55	823.17
SEm ±	0.78	1.98	9.27	10.45	5.67	9.25
LSD (*p* = 0.05)						
Weed management						
Sulfosulfuron + carfentrazone (25 + 20 g/ha) at 30–35 DAS	43.22	125.54	485.54	484.08	243.48	915.71
Clodinafop-propargyl + metsulfuron (60 + 4 g/ha) at 30–35 DAS	41.98	124.06	476.92	477.29	236.33	906.06
Clodinafop-propargyl + metribuzin (54 + 120 g/ha) at 30–35 DAS	40.15	118.56	455.04	456.33	229.56	898.94
Control	36.19	113.62	365.96	340.08	206.50	765.13
SEm ±	0.54	2.04	8.69	10.52	4.22	9.06
LSD (*p* = 0.05)	1.88	7.07	30.07	36.41	14.61	31.34
Interaction	NS	NS	NS	NS	NS	NS

Treatment means were compared at *P* ≤ 0.05 level using least significant difference (LSD).

**Table 5:** Effect of nitrogen, residue and weed management practices on yield attributes and yield of wheat (Average of 2018–2019 & 2019–2020).

Treatments	Spike m^−2^	Grains spike^−1^	1000 grain weight (g)	Grain yield (kg ha^−1^)	Straw yield (kg ha^−1^)	Harvest index
Nitrogen and residue management						
100% RDN* + Rice residue	411.58	34.25	36.71	3804.21	5433.10	43.66
125% RDN + Rice residue	452.17	36.96	38.58	4238.13	5970.06	44.57
125% RDN + Rice residue + Waste decomposer (WD)	457.50	36.92	38.68	4267.42	6010.32	44.88
100% RDN	399.33	33.04	36.46	3518.46	5228.63	43.69
SEm ±	9.93	0.49	0.73	102.46	140.83	0.84
LSD (*p* = 0.05)	34.37	1.71	NS	354.54	487.32	NS
Weed management						
Sulfosulfuron + carfentrazone (25 + 20 g/ha) at 30–35 DAS	477.88	37.75	39.16	4347.88	6162.32	44.13
Clodinafop-propargyl + metsulfuron (60 + 4 g/ha) at 30–35 DAS	469.75	36.50	38.27	4269.08	6032.05	44.53
Clodinafop-propargyl + metribuzin (54 + 120 g/ha) at 30–35 DAS	454.00	35.54	37.67	4137.33	5988.15	44.34
Control	318.96	31.38	35.32	3073.92	4459.60	43.80
SEm ±	14.30	0.43	0.55	61.69	139.61	0.70
LSD (*p* = 0.05)	49.49	1.49	1.90	213.48	483.13	NS
Interaction	NS	NS	NS	NS	NS	NS

Treatment means were compared at *P* ≤ 0.05 level using least significant difference (LSD).

Similarly, concerning yield attributes and yield, 125% RDN + R + WD showed significantly higher spike m^−2^, grains spike^−1^, grain, and straw yields over 100% RDN + R and 100% RDN, but it was statistically at par with 125% RDN + R. While, 1000-grain weight and harvest index were not significantly influenced by residue management practices. Additional, 125% RDN + R + WD, 125% RDN + R and 100% RDN + R produced 17.55, 16.98, and 7.41% higher grain yields over control treatment, respectively. However, among weed management practices, all herbicidal treatment produced significantly higher yield attributes viz., spike m^−2^, and grains spike^−1^ and 1000-grain weight as well as grain and straw yield than that of control treatments. Further, sulfosulfuron + carfentrazone observed significantly higher yield components, grain and straw yields as compared to control but remained statistically at par with clodinafop-propargyl + metsulfuron and clodinafop-propargyl + metribuzin. Nevertheless, a reduction of 29.30, 28.00 and 25.70% in grain yield was observed in control treatment compared to sulfosulfuron + carfentrazone, clodinafop-propargyl + metsulfuron and clodinafop-propargyl + metribuzin, respectively.

### Energy budgeting

#### Energy use pattern

**Figure 1:**
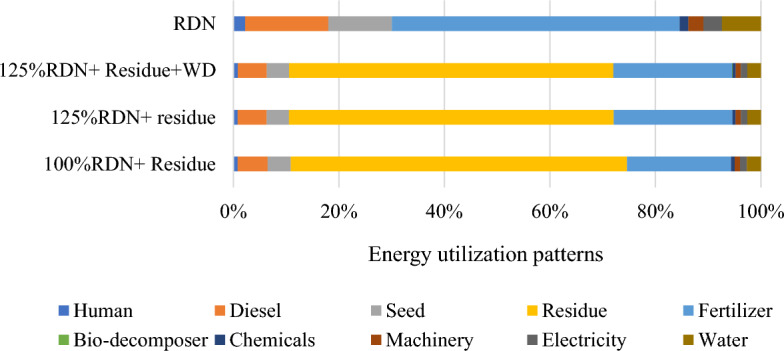
Source wise energy utilization patterns among residue management practices.

**Figure 2:**
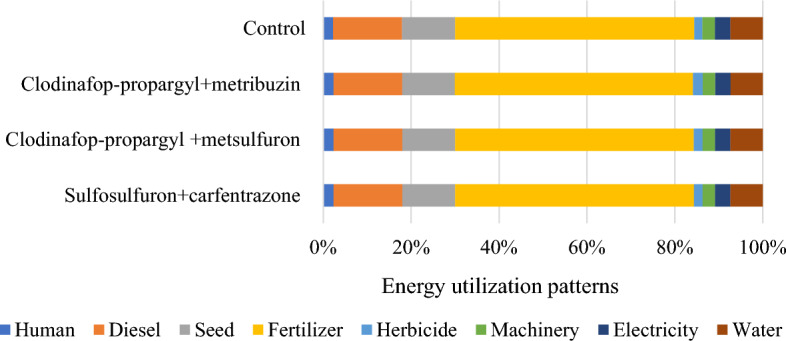
Source wise energy utilization patterns among different weed management practices.

**Figure 3:**
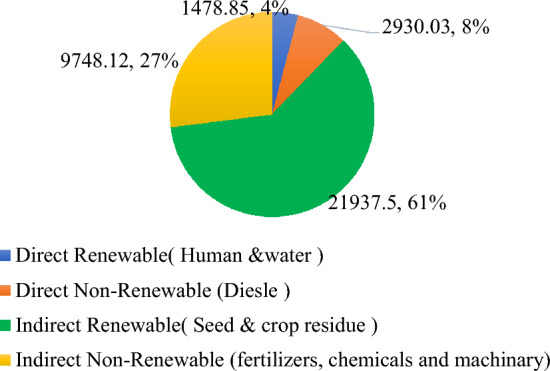
Renewable and non-renewable energy sources among residue management practices.

**Figure 4:**
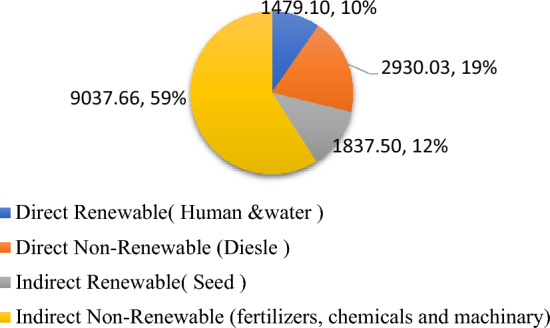
Renewable and non-renewable energy sources among weed management practices.

The source and operation-wise energy use pattern was computed for residue and weed management practices (Figs. 1 and 2). In general, in residue management, indirect renewable energy namely crop residue and seed contributed the highest input energy 61%, followed by indirect non-renewable energy namely fertilizers, chemicals and machinery 27%, direct non-renewable namely diesel and electricity 8% and direct renewable namely human and water 4% (Fig. 3). While, in weed management practices, indirect non-renewable energy namely fertilizers, chemicals and machinery consumed highest energy input 59%, followed by direct non-renewable namely diesel and electricity 19%, indirect renewable (seed) 12% and direct renewable namely human and water 10% (Fig. 4.) Besides, among residue retention treatments 125% RDN + R + WD and 125% RDN + R equally exhibited higher total energy input consumption followed by 100% RDN + R and the lest total energy input was utilized in 100% RDN without residue. Among weed management practices, the order of energy input unitization was clodinafop-propargyl + metribuzin > clodinafop-propargyl + metsulfuron > sulfosulfuron + carfentrazone and control treatment.

#### Energy input and output relationship

On an average, the highest amount of energy was accumulated in wheat straw as compared to grain in both residue and weed management practices. The energy accumulation in seeds was 45%, the remaining 55% was accumulated in straw. Furthermore, 125% RDN + R + WD observed significantly higher energy output (137,860 MJ ha^−1^) as compared to 100% RDN + R residue and 100% RDN but was statistically at par with 125% RDN + R. (Table 6). Moreover, 125% RDN + R + WD, 125% RDN + R and 100% RDN found to be statistically at par with each other but recorded significantly higher output energy as compare to 100% RDN + R. Besides, 100% RDN recorded significantly higher energy use efficiency, energy productivity and energy profitability as compared to all treatments. With regard to weed management practices, all herbicidal treatment was found to be statistically at par with each other but recorded significantly higher energy output, net energy, energy use efficiency, productivity and profitability as compared to control treatment. However, sulfosulfuron + carfentrazone observed numerically higher energy output (140,942 MJ ha^−1^), net energy (104,778 MJ ha^−1^), energy use efficiency (4.70), energy productivity (0.14) and energy profitability (3.70) which was followed by clodinafop-propargyl + metsulfuron and clodinafop-propargyl + metribuzin and the lest energy indices were recorded by control treatment (Table 7).

**Table 6:** Effect of nitrogen, residue and weed management practices on grain yield, energy-on-energy output of wheat (Average of 2018–2019 & 2019–2020).

Treatment	Grain yield (kg ha^−1^)	Grain yield energy output (MJ ha^−1^)	Straw yield (kg ha^−1^)	Straw yield energy output (MJ ha^−1^)	Total energy output (MJ ha^−1^)	Percentage total output increase over control
Nitrogen and residue management						
100% RDN* + Rice residue	3804.21	55,921.86	5433.10	67,913.80	123,266.24	5.46
125% RDN + Rice residue	4238.13	62,300.44	5970.06	74,625.69	135,963.72	14.49
125% RDN + Rice residue + Waste decomposer (WD)	4267.42	62,731.03	6010.32	75,129.01	136,914.81	15.07
100% RDN	3518.46	51,721.34	5228.63	65,357.93	116,908.29	
SEm ±	102.46	1506.10	140.83	1760.32	2497.13	
LSD (*p* = 0.05)	354.54	5211.79	487.32	6091.52	8641.22	
Weed management						
Sulfosulfuron + carfentrazone (25 + 20 g/ha) at 30–35 DAS	4347.88	63,913.76	6162.32	77,028.97	140,019.18	28.39
Clodinafop-propargyl + metsulfuron (60 + 4 g/ha) at 30–35 DAS	4269.08	62,755.53	6032.05	75,400.56	137,437.14	26.94
Clodinafop-propargyl + metribuzin (54 + 120 g/ha) at 30–35 DAS	4137.33	60,818.80	5988.15	74,851.84	134,921.92	25.61
Control	3073.92	45,186.58	4459.60	55,745.05	100,674.81	–
SEm ±	61.69	906.85	139.61	1745.17	1847.23	–
LSD (*p* = 0.05)	213.48	3138.13	483.13	6039.07	6392.27	–
Interaction	NS	NS	NS	NS	NS	–

Treatment means were compared at *P* ≤ 0.05 level using least significant difference (LSD).

**Table 7:** Effect of nitrogen, residue and weed management practices on energy indices of wheat (Average of 2018–2019 & 2019–2020).

Treatment	Energy input (MJ ha^−1^)	Energy output (MJ ha^−1^)	Net energy (MJ ha^−1^)	Energy use efficiency	Energy productivity (kg MJ^−1^)	Energy profitability (kg MJ^−1^)
Nitrogen and residue management						
RDN + R	42,084.29	123,835.66	81,751.37	2.94	0.09	1.94
125% RDN + R	43,614.97	136,926.13	93,311.16	3.14	0.10	2.14
125% RDN + R + WD	43,688.45	137,860.04	94,171.59	3.16	0.10	2.16
RDN	15,277.00	117,079.26	101,802.26	7.66	0.23	6.66
SEm ±	–	2480.83	2480.83	0.10	0.00	0.10
LSD (*p* = 0.05)	–	8584.79	8584.79	0.33	0.01	0.33
Weed management						
Sulfosulfuron + carfentrazone (25 + 20 g/ha) at 30–35 DAS	36,164.18	140,942.73	104,778.56	4.70	0.14	3.70
Clodinafop-propargyl + metsulfuron (60 + 4 g/ha) at 30–35 DAS	36,176.20	138,156.09	101,979.89	4.54	0.14	3.54
Clodinafop-propargyl + metribuzin (54 + 120 g/ha) at 30–35 DAS	36,183.38	135,670.64	99,487.26	4.37	0.13	3.37
Control	36,140.95	100,931.62	64,790.68	3.29	0.10	2.29
SEm ±	–	1934.67	1934.67	0.04	0.00	0.04
LSD (*p* = 0.05)	–	6694.83	6694.83	0.15	0.01	0.15
Interaction	–	NS	NS	NS	NS	NS

Treatment means were compared at *P* ≤ 0.05 level using least significant difference (LSD).

#### Carbon budgeting

**Figure 5:**
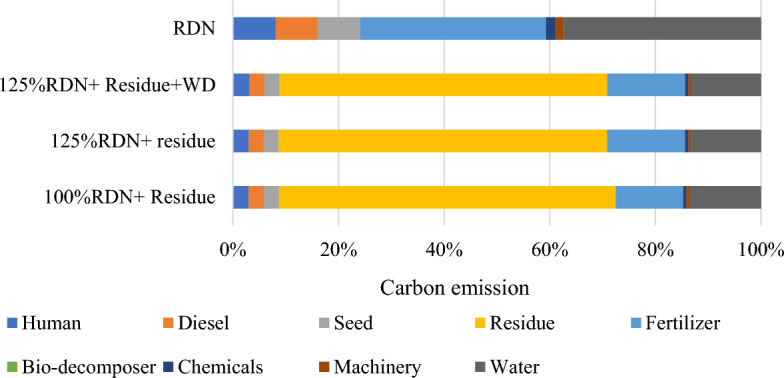
Source wise carbon emission patterns among different residue management practices.

**Figure 6:**
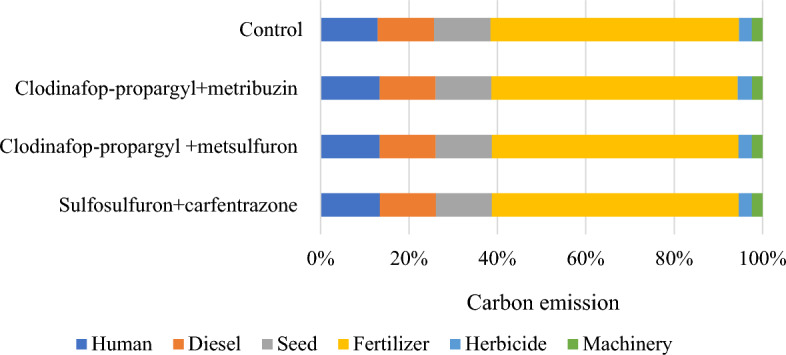
Source wise carbon emission patterns among different weed management practices.

With respect to residue management practices, among various sources rice residue consumed 56.03% of total carbon input, followed by fertilizers 16.34%, water 15.88% and diesel 3.39% and the remaining 8.39% was consumed by human, seeds, chemicals and machinery (Fig. 5). However, among weed management practices, water consumed 37.25% of total carbon input followed by fertilizer 35.09%, human 8.33%, diesel 7.97%, seed 7.96% and the remaining sources namely herbicide and machinery consumed only 3.41% carbon input (Fig. 6). Hence, 125% RDN + R + WD recorded significantly higher carbon output as compared to 100% RDN + R and 100% RDN but was statistically at par with 125% RDN + R. However, carbon efficiency and carbon footprint was significantly higher under 100% RDN without residue treatment as compared to all residue retention treatments. Among weed management, sulfosulfuron + carfentrazone observed significantly higher carbon output, carbon efficacy and carbon footprint as compared to control but was statically at par with clodinafop-propargyl + metsulfuron and clodinafop-propargyl + metribuzin (Table 8).

**Table 8:** Effect of nitrogen, residue and weed management practices on carbon indices of wheat (Average of 2018–2019 & 2019–2020).

Treatment	Total carbon input (kg CE ha^−1^)	Total carbon output (kg CE ha^−1^)	Carbon efficiency	Carbon footprint (kg CE kg^−1^ wheat)
Nitrogen and residue management				
RDN + R	1382.24	4064.42	2.94	0.37
125% RDN + R	1416.58	4491.60	3.17	0.34
125% RDN + R + WD	1418.50	4522.20	3.19	0.34
RDN	502.24	3848.72	7.66	0.15
SEm ±	–	81.86	0.09	0.01
LSD (*p* = 0.05)	–	283.28	0.33	0.03
Weed management				
Sulfosulfuron + carfentrazone (25 + 20 g/ha) at 30–35 DAS	1180.21	4624.48	4.71	0.27
Clodinafop-propargyl + metsulfuron (60 + 4 g/ha) at 30–35 DAS	1180.33	4532.50	4.56	0.27
Clodinafop-propargyl + metribuzin (54 + 120 g/ha) at 30–35 DAS	1181.02	4455.21	4.38	0.28
Control	1178.00	3314.75	3.31	0.38
SEm ±	–	66.17	0.05	0.01
LSD (*p* = 0.05)	–	228.98	0.17	0.15
Interaction	–	NS	NS	NS

Treatment means were compared at *P* ≤ 0.05 level using least significant difference (LSD).

#### Economics analysis

The production cost and economic analysis of different residue and weed management are shown in Table 9. Among residue management practices, higher cost of cultivation was observed in 125% RDN + R + WD which was followed by 125% RDN + R, 100% RDN + R and 100% RDN without residue. While among weed management practices, higher cost of cultivation was recorded by clodinafop-propargyl + metasulfuron which was closely followed by sulfosulfuron + carfentrazone and clodinafop-propargyl + metribuzin and lowest cost of production was observed in control treatment. The highest gross returns (85,889 ₹./ha), net returns (60,160 ₹./ha) and benefit cost ratio (2.34) was observed in 125% RDN + R + WD which was closely followed by 125% RDN + R. The treatment 100% RDN without residue recorded lowest gross returns, net returns and benefit cost ratio. Concerning weed management practices, sulfosulfuron + carfentrazone observed maximum gross returns, net returns and benefit cost ratio which was followed by clodinafop-propargyl + metsulfuron and clodinafop-propargyl + metribuzin. While control treatment recorded lowest gross returns, net returns and benefit cost ratio among all treatments.

**Table 9:** Effect of nitrogen, residue and weed management practices on relative economics of treatments (Average of 2018–2019 & 2019–2020).

Treatment	Gross returns (₹. Ha^−1^)	Cost of cultivation (₹ ha^−1^)	Net returns (₹. ha^-1^)	Benefit cost ratio
Nitrogen and residue management				
RDN + R	76,571.5	25,379.5	51,192.0	2.02
125% RDN + R	85,300.5	25,679.5	59,621.0	2.32
125% RDN + R + WD	85,889.0	25,729.5	60,160.0	2.34
RDN	70,825.5	25,379.5	45,446.0	1.79
Weed management				
Sulfosulfuron + carfentrazone (25 + 20 g/ha) at 30–35 DAS	87,511.5	26,055	61,456.5	2.37
Clodinafop-propargyl + metsulfuron (60 + 4 g/ha) at 30–35 DAS	85,926.0	26,430	59,496.0	2.26
Clodinafop-propargyl + metribuzin (54 + 120 g/ha) at 30–35 DAS	83,274.0	25,652	57,622.0	2.26
Control	61,875.0	24,030	37,845.0	1.58

#### Soil studies

The data with respect to soil properties namely soil organic carbon, available NPK and bulk density are shown in Table 10. Both residue and weed management practices did not had significant effect on soil nutrient status. But among residue management the magnitude of increase in soil nutrient status namely soil organic carbon, available nitrogen, phosphorus and potassium with residue bearing treatments ranged from 3.2–4.39%, 1.84–4.36%, 3.75–7.6% and 1.46–2.74%, respectively over no residue treatments. While among weed management practices, all herbicidal treatment almost recorded similar soil nutrient status.

**Table 10:** Effect of nitrogen, residue and weed management practices on soil properties in wheat (Average of 2018–2019 & 2019–2020).

Treatments	OC (g kg^−1^)	Available N (kg ha^−1^)	Available P (kg ha^−1^)	Available K (kg ha^−1^)	Bulk density (g cc^-1^)
Nitrogen and residue management					
100% RDN* + Rice residue	4.05	233.23	14.38	144.45	1.45
125% RDN + Rice residue	4.06	237.70	14.82	145.68	1.45
125% RDN + Rice residue + Waste decomposer (WD)	4.10	239.37	14.98	146.36	1.44
100% RDN	3.94	232.16	13.86	141.83	1.46
SEm ±	0.10	4.86	0.29	1.95	0.01
LSD (*p* = 0.05)	NS	NS	NS	NS	NS
Weed management					
Sulfosulfuron + carfentrazone (25 + 20 g/ha) at 30–35 DAS	4.03	232.15	14.03	141.71	1.45
Clodinafop-propargyl + metsulfuron (60 + 4 g/ha) at 30–35 DAS	3.99	233.95	14.35	143.96	1.45
Clodinafop-propargyl + metribuzin (54 + 120 g/ha) at 30–35 DAS	4.03	236.70	14.62	144.18	1.46
Control	4.11	239.67	15.05	148.48	1.45
SEm ±	0.11	2.55	0.40	3.32	0.02
LSD (*p* = 0.05)	NS	NS	NS	NS	NS
Initial	3.92	228.93	13.84	142.34	1.46

## Discussion

### Effect of residue and weed management practices on weed density and biomass

The current study found that zero tillage with standing rice residue treatments observed significantly less weed density and biomass than no residue treatment. This is in conformity with results of Kumar et al.^17^, and Nath et al.^18^ both observed lower weed density and biomass in zero tillage with residue retention. In addition, Nandan et al.^19^ found that sites managed with agricultural residue retention caused decreased weed species richness. Besides, according to Teasdale et al.^20^ agricultural residues retention on the surface of soil influenced the physiochemical environment of seed emergence which resulted in lower density of weeds. Furthermore, Nesar et al.^21^ also reported that the reduction in weed population and biomass in paddy straw containing treatments may happen as result of paddy straw on the surface of soil which helps to reduce weed seed emergence by avoiding light exposure and mechanical impedance of weed seedlings. On the other hand, crop residue on the soil surface limits light, preventing weed seed germination and growth. Henceforth, less grassy and broad-leaved density and biomass under residue retained treatments may be owing to rice residue, which may inhibited weed germination by blocking light and containing phytotoxic chemicals, resulting in lower weed density. Correspondingly, Susha et al.^22^ also found that zero tillage with residue treatments reduced broad-leaved and narrow-leaved weeds, confirming the smothering impact of rice residue on weeds. In addition, the results of our research showed that weed density and biomass in herbicidal treatments were significantly declined as compared to control treatment. Addition, the combination of clodinafop-propargyl + metasulfuron found more effective in controlling weed density as well as weed biomass. These results are comparable to those found by Rani et al.^23^. Likewise, Kumar et al.^17^ found that the use of metsulfuron at a rate of 4 g ha^−1^ combined with clodinafop at a rate of 60 g ha^−1^ resulted in lower density and biomass of grassy weeds.

### Effect of residue and weed management practices on wheat growth, yield attributes and yields of wheat

The current research also disclosed that 125% RDN + R with or without waste decomposer provided significantly higher growth parameter. This is also in agreement with findings of Ali et al.^24^ who also reported that residue retention with 25% additional N enhanced wheat growth parameters than residue retention with 100% RDN. The superior growth indices with standing rice residue along with 125% RDN with or without waste decomposer could be due to due to better soil moisture conducive for good germination which brought good crop establishment and supported growth parameters in residue retention treatment^24,25^. On the other hand, applying a higher amount of nitrogen to standing rice residue may promote the process mineralization, resulting in gradual release of nutrients in the soil solution, increased nutrient availability to wheat crop, and increased nutrient uptake, which accelerated wheat growth parameters. With respect to herbicidal treatments, it found that wheat growth parameters were significantly greater in herbicidal treatments than in the control treatment. This could be because of more efficacy of herbicide in reducing the number of weeds and, in turn, the degree of competition between the crop and the weeds for resources and light. This, in turn, led to superior growth parameters, including superior plant height, tillers/m^2^, and plant dry matter.

In a similar vein, present research outcome also revealed that the wheat yield attributes namely no. of spikes m^−2^, grains spike^−1^ as well as grain and straw yields were significantly increased by standing rice residue plus 125% RDN with or without waste decomposer as opposed to the no residue treatment. Therefore, 125% RND along with residue with or without waste decomposer produced significantly higher yield attributes namely number of grains spike^−1^, grain yield, and straw yields. The beneficial effect of crop residues on soil physicochemical and microbiological properties, in particular soil moisture, nutrient availability, and temperature modulation, may be responsible for these findings^26,27^. In a similar fashion, Kumar et al.^28^ mentioned that wheat sown with pigeonpea together resulted in higher grain and straw production when they used treatments that included residue retention as opposed to treatments that did not include residue. In terms of growth and yield, 125% RDN + R with or without waste decomposer performed statistically similar. This could be owing to the fact that the waste decomposer used on standing rice residue was designed for the breakdown of organic waste and residue heaps, and its performance may differ from that of standing rice residue in an open field. On the other hand, the low temperature at the trial site could be another reason why the waste decomposer did not function effectively.

### Effect of residue and weed management practices on energy indices and carbon footprints

In terms of different energy indices, all residue retention treatments observed considerably more energy input than that of no residue. The rice straw accounted for 58.61% of the total energy input, followed by fertilizer with 26.40%. This is consistent with the findings of Kumar et al.^28^, Lal el et al.^29^ and Choudhary et al.^30^ who also found increased energy consumption as a result of application residue in pigeonpea-wheat cropping systems, rice-maize cropping systems, and millet-mustard cropping systems, respectively. Furthermore, 100% RDN with no residue obtained significantly higher net energy, energy use efficiency, energy productivity and energy profitability which was due to no use of rice residue and consumption of less energy input which was also confirmed by Saad et al.^31^, and Kumar et al.^28^. The energy indices for all the herbicides were significantly greater than those for the control. In addition, sulfosulfuron + carfentrazone obtained considerably greater production, net energy, energy usage efficiency, and energy productivity when compared to clodinafop-propargyl + metsulfuron and clodinafop-propargyl + metribuzin, respectively. Higher energy output and other energy indices under herbicidal treatments could be attributable to the higher efficacy of herbicides on weed suppression, which reduced weed-crop competition. As a result, crop unitization of available sources such as nutrients, water, light, and space property led to higher grain and biomass production.

The largest overall carbon input was noticed in residue retention treatments with thick residue coverage. Crop residue includes 44% carbon input; hence 125% RDN + R + WD has a greater carbon input^32,33^. Among residue management, rice residue contributed 56.03% of carbon input, followed by fertilizer (16.31%). In weed control, water and fertilizer contributed 37.25% and 35.09%, respectively. 125% RDN + R + WD had significantly higher carbon input than control and 100% RDN + R but was found to be statistically equal to 125% RDN + R due to higher grain and biomass output. As no residue was used, 100% RDN had a much greater carbon efficiency (7.66) and a lower carbon footprint (0.15 kg CE kg^−1^ wheat), which is also in agreement with the findings of Zhanga et al.^34^. Sulfosulfuron + carfentrazone weed control has increased carbon output, efficiency, and footprint. This is due to higher grain and straw yields as a result of higher efficacy on controlling weeds.

### Effect residue and weed management on economics of wheat

The data depicted in Table 9 revealed that under 125% RDN + R + WD, gross returns, net returns, and benefit cost ratio were all higher than 100% RDN + R and 100% RDN. The beneficial effect of crop residue mulching under various residue retention treatments, which significantly improved yield attributes and yield, may be responsible for this monetary variance^35,36^. Additionally, due to fewer weed completion and higher yield components, sulfosulfuron + carfentrazone was shown to have a much higher cost of production, gross returns, net returns, and benefit cost ratio, followed by clodinafop-propargyl + metsulfuron and clodinafop + metribuzin. Heavy weed infestation may have drained resources and led to subpar agricultural production, explaining why economic indicators improved less under treatment.

## Conclusion

Based on two years research it concluded that all residue retention treatments noticed lower density and biomass of grassy and broad-leaved weeds than treatments with no residue. Nevertheless, 125% RDN + R + WD and 125% RDN + R recorded considerably higher growth parameters, yield components, grain yield and straw yield as compared to 100% RDN + R and 100% RDN without residue. In a similar vein, 125% RDN + R + WD recorded higher energy output, carbon output, gross returns, net returns, and the benefit cost ratio. Further 100% RDN without residue obtained significantly higher net energy, energy use efficiency, energy profitability, and energy productivity, as well as carbon efficiency, and lower carbon footprint in comparison to the other treatments, but it produced significantly lower levels of grain yield, straw yield, gross returns, and net returns in comparison to the other treatments. When it came to the effectiveness of herbicidal weed management measures, all three herbicides performed similar to each other, although they recorded meaningfully lower density and biomass in comparison to the control treatment. Among the herbicidal treatment, clodinafop-propargyl + metasulfuron showed significantly higher growth, yield, energy output, energy use efficiency, net energy, energy profitability, carbon output, and a less carbon footprint in comparison to the control, but it was statistically comparable to clodinafop + metribuzin and clodinafop-propargyl + metasulfuron.

Therefore, based on present study it can be recommended that rice residue retention with 25% additional nitrogen and weed management by clodinafop-propargyl + metasulfuron herbicide found suitable for zero tillage wheat (Supplementary material).

### Supplementary Information

Supplementary Information.

## Data Availability

All data generated or analysed during this study were included in the article. The raw data were provided as supporting file. (We confirmed the data used to support the findings of this study were available from the corresponding author upon request).
