# Supplementary material for: Effect of residue and weed management practices on weed flora, yield, energetics, carbon footprint, economics and soil quality of zero tillage wheat
Source: Sci Rep. 2023 Nov 7;13:19311. doi: 10.1038/s41598-023-45488-3 (PMC10630349; doi:10.1038/s41598-023-45488-3)
Supplement: Supplementary file 1 — Supplementary Information. [file 41598_2023_45488_MOESM1_ESM.docx]

**Table 1: Physiochemical properties of soil and methods used for analysis**

| S. No | Soil parameters | Value | Methods and Reference |
| --- | --- | --- | --- |
| 1 | Soil pH | 7.46 | 1:2.5 Soil water suspension measured with Glass electrode pH meter (Jackson^1^) |
| 2 | Soil EC | 0.16 | 1:2.5 Soil water suspension measured with conductivity meter (Jackson^1^) |
| 3 | OC (g kg^-1^) | 3.92 | Dichromate oxidatioan of organic matter  (Jackson^1^) |
| 4 | Available Nitrogen (kg ha^-1^) | 288.93 | Alkaline Potassium Permanganate method (Subbiah and Asija^2^) |
| 5 | Available phosphorus (kg ha^-1^) | 13.84 | Sodium Bicarbonate method (Olsen et al^3^) |
| 6 | Available potassium (kg ha^-1^) | 142.34 | Ammonium acetate method (Jackson^1^) |
| 7 | Bulk density (g m^-2^) | 1.46 | Blake and Hartge^4^ |

**Table 2: Energy equivalent for various Agricultural Inputs/ Outputs under zero tillage wheat**

| Particulars | Units | Equivalent energy (MJ) | Reference |
| --- | --- | --- | --- |
| Input | | |  |
| Machinery | | | |
| Machinery: seed drill | h | 25.08 | Choudhary et al^5^ |
| Knapsack sprayer | h | 0.17 | Dagistan et al^6^ |
| Electricity | KWh | 11.93 | Mittal and Dhawan^7^ |
| Diesel | L | 56.31 | Devasenapathy et al^8^ |
| Irrigation water | m^3^ | 1.02 | Singh et al^9^ |
| Human power | Man-hour | 1.96 | Choudhary et al^5^ |
| Fertilizer | | | |
| N | kg | 60.6 | Mittal and Dhawan^7^ |
| P_2_O_5_ | kg | 11.1 | Choudhary et al^5^ |
| K_2_O | kg | 6.7 | Choudhary et al^5^ |
| Chemicals | | | |
| Herbicide | kg a.i | 288 | West and Marland^10^ |
| Seed | kg | 14.7 | Devasenapathy et al^8^ |
| Rice Residue | kg | 12.5 |  |
| Output | | | |
| Wheat grain | kg | 14.7 | Choudhary et al^5^ |
| Wheat straw | kg 12.5 | kg 12.5 | Devasenapathy et al^8^ |

**Table 3: Energy equivalent for various Agricultural Inputs/ Outputs under zero tillage wheat**

| Particulars | Units | Equivalent carbon emission  (kg CE kg^-1^) | Reference |
| --- | --- | --- | --- |
| Input |  |  |  |
| Machinery | h | 0.89 | Lal et al^11^ |
| Diesel | L | 0.94 | Lal et al^11^ |
| Seed | kg | 0.32 | Wang et al^12^ |
| Nitrogen | kg | 1.3 | Deng^13^ |
| Phosphorus | kg | 0.2 | Deng^13^ |
| Potash | kg | 0.15 | Deng^13^ |
| Glyphosate | kg | 9.1 | Lal et al^11^ |
| Human labour | Man hour | 0.23 | Lal et al^11^ |
| Irrigation water | m^3^ | 0.17 | Liu et al^14^  Houshyar et al^15^ |
| Rice residue | kg | 0.44 | Choudhary et al^5^ |

**Table 4. Fixed energy equivalent**

| Particulars | Units | Quantity | Total |
| --- | --- | --- | --- |
| Input | | | |
| Diesel | L | 56.32 | 2393.175 |
| Seed | kg | 125 | 1837.5 |
| Nitrogen | kg | 125 | 7575 |
| Phosphorus | kg | 50 | 555 |
| Potash | kg | 25 | 167.5 |
| Glyphosate | kg | 1 | 288 |
| Fertilizer application | Man hour | 56 | 109.76 |
| Irrigation | m3 | 1100 | 1122 |
| Electricity | kwh | 45 | 536.85 |
| sowing (seed drill) | h | 3.5 | 87.78 |
| Harvesting | man hour | 120 | 235.2 |
| Threshing | h | 5 | 344 |
| Total | | | 15251.77 |

**Table 5. Variable energy input**

|  | Particulars | Units | Quantity | Total |
| --- | --- | --- | --- | --- |
| Nitrogen and residue management | | | | |
| RDN | Rice residue | kg | 2000 | 26800 |
|  | Total |  | | 26800 |
| RDN + 25 % N | Rice residue | kg | 2000 | 26800 |
|  | Nitrogen | 25 | 60.6 | 1515 |
|  | Labour | 8 | 1.96 | 15.68 |
|  | Total |  | | 28330.68 |
| RDN + 25 % N+ waste decomposer | Rice residue | 2000 | 12.5 | 26800 |
|  | Nitrogen | 25 | 60.6 | 1515 |
|  | WD | L | 5 | 50 |
|  | Water | m^3^ | 0.5 | 0.51 |
|  | labour | 16 | 1.96 | 31.36 |
|  | Total |  | | 28396.87 |
| RDN | - | - | -- | - |
| Weed management | | | | |
| Sulfosulfuron+carfentrazone (25 + 20 g/ha) at 30-35 DAS | Sulfo+Carfe | kg | 0.045 | 12.96 |
|  | Water | m3 | 0.5 | 0.51 |
|  | labour | Man hour | 8 | 15.68 |
|  | Total |  | | 29.15 |
| Clodinafop-propargyl +metsulfuron (60 +4 g/ha) at 30-35 DAS | Clodino+Met | kg | 0.064 | 18.432 |
|  | Water | m3 | 0.5 | 0.51 |
|  | labour | Man hour | 8 | 15.68 |
|  | Total |  | | 34.62 |
| Clodinafop-propargyl+metribuzin (54 +120 g/ha) at 30-35 DAS | clodino+Metri | kg | 0.174 | 50.112 |
|  | Water | m3 | 0.5 | 0.51 |
|  | Labour | 15.68 | 8 | 15.68 |
|  | Total |  |  | 66.30 |
| Control | - | - | - | - |

**Table 6. Total energy equivalent input**

| Treatment details | Fixed energy | N + Residue | Weed management | Total |
| --- | --- | --- | --- | --- |
| 100% RDN+ Paddy residue +Sulfosulfuron+carfentrazone (25 + 20 g/ha) at 30-35 DAS | 15251.77 | 26800 | 29.15 | 42080.92 |
| 100% RDN+ Paddy residue + Clodinafop-propargyl +metsulfuron (60 +4 g/ha) at 30-35 DAS | 15251.77 | 26800 | 34.62 | 42086.39 |
| 100% RDN+ Paddy residue +Clodinafop-propargyl+metribuzin (54 +120 g/ha) at 30-35 DAS | 15251.77 | 26800 | 66.30 | 42118.07 |
| 100% RDN+ Paddy residue + No herbicide | 15251.77 | 26800 | 0 | 42051.77 |
| 125% RDN+ Paddy residue +Sulfosulfuron+carfentrazone (25 + 20 g/ha) at 30-35 DAS | 15251.77 | 28330.68 | 29.15 | 43611.6 |
| 125% RDN+ Paddy residue + Clodinafop-propargyl +metsulfuron (60 +4 g/ha) at 30-35 DAS | 15251.77 | 28330.68 | 34.62 | 43617.07 |
| 125% RDN+ Paddy residue +Clodinafop-propargyl+metribuzin (54 +120 g/ha) at 30-35 DAS | 15251.77 | 28330.68 | 66.30 | 43648.75 |
| 125% RDN+ Paddy residue + No herbicide | 15251.77 | 28330.68 | 0 | 43582.45 |
| 125% RDN+ Paddy residue + waste decomposer +Sulfosulfuron+carfentrazone (25 + 20 g/ha) at 30-35 DAS | 15251.77 | 28396.87 | 29.15 | 43677.79 |
| 125% RDN+ Paddy residue +waste decomposer+ Clodinafop-propargyl +metsulfuron (60 +4 g/ha) at 30-35 DAS | 15251.77 | 28396.87 | 34.62 | 43683.26 |
| 125% RDN+ Paddy residue +waste decomposer+Clodinafop-propargyl+metribuzin (54 +120 g/ha) at 30-35 DAS | 15251.77 | 28396.87 | 66.30 | 43714.94 |
| 125% RDN+ Paddy residue + waste decomposer +No herbicide | 15251.77 | 28396.87 | 29.15 | 43677.79 |
| RDN+Sulfosulfuron+carfentrazone (25 + 20 g/ha) at 30-35 DAS | 15251.77 | 0 | 34.62 | 15286.39 |
| RDN+Clodinafop-propargyl +metsulfuron (60 +4 g/ha) at 30-35 DAS | 15251.77 | 0 | 66.30 | 15318.07 |
| RDN+Clodinafop-propargyl+metribuzin (54 +120 g/ha) at 30-35 DAS | 15251.77 | 0 | 0 | 15251.77 |
| RDN+ No herbicide | 15251.77 | 0 | 0 | 15251.77 |

**Table 7: Energy equivalent for various Agricultural Inputs/ Outputs under zero tillage wheat**

| Particulars | Units | Equivalent carbon emission (kg CEkg^-1^) | Reference |
| --- | --- | --- | --- |
| Input | | | |
| Machinery | h | 0.89 | Lal^16^ |
| Diesel | L | 0.94 | Lal^16^ |
| Seed | kg | 0.32 | Lal^16^ |
| Nitrogen | kg | 1.3 | Lal^16^ |
| Phosphorus | kg | 0.2 | Lal^16^ |
| Potash | kg | 0.15 | Lal^16^ |
| Glyphosate | kg | 9.1 | Lal^16^ |
| Human labour | Man hour | 0.23 | Lal^16^ |
| Irrigation | m^3^ | 0.17 | Lal^16^ |
| Rice residue | kg | 0.44 | Lal^16^ |

**Table 8. Fixed energy equivalent**

| Particulars | Units | Quantity | Total |
| --- | --- | --- | --- |
| Diesel | L | 42.5 | 39.95 |
| Seed | kg | 125 | 40 |
| Nitrogen | kg | 125 | 162.5 |
| Phosphorus | kg | 50 | 10 |
| Potassium | kg | 25 | 3.75 |
| Glyphosate | kg | 1 | 9.1 |
| Fertilizer and herbicide application | Man hour | 56 | 12.88 |
| Irrigation | m3 | 1100 | 187 |
| Sowing (Seed Drill) | kwh | 3.5 | 3.115 |
| Harvesting | h | 120 | 27.6 |
| Threshing | man hour | 5 | 4.45 |
| Total |  |  | 15251.77 |

**Table 9. Variable energy input**

|  | Particulars | Units | Quantity | Total |
| --- | --- | --- | --- | --- |
| Nitrogen and residue management | | | | |
| RDN | Rice residue | kg | 0.44 | 880 |
|  | Total |  | | 880 |
| RDN + 25 % N | Rice residue | kg | 0.44 | 880 |
|  | Nitrogen | kg | 1.3 | 32.5 |
|  | Labour | Man hour | 0.23 | 1.84 |
|  | Total |  | | 914.34 |
| RDN + 25 % N+ waste decomposer | Rice residue | 2000 | 0.44 | 880 |
|  | Nitrogen | 25 | 1.3 | 32.5 |
|  | Water | 0.5 | 0.17 | 0.085 |
|  | labour | 16 | 0.23 | 3.68 |
|  | Total |  | | 916.265 |
| RDN | - | - | -- | - |
| Weed management | | | | |
| Sulfosulfuron+carfentrazone (25 + 20 g/ha) at 30-35 DAS | Sulfo+Carfe | kg | 0.045 | 0.28 |
|  | Water | m3 | 0.5 | 0.09 |
|  | labour | Man hour | 8 | 1.84 |
|  | Total |  | | 2.21 |
| Clodinafop-propargyl +metsulfuron (60 +4 g/ha) at 30-35 DAS | Clodino+Met | kg | 0.064 | 0.4032 |
|  | Water | m3 | 0.5 | 0.09 |
|  | labour | Man hour | 8 | 1.84 |
|  | Total |  | | 34.62 |
| Clodinafop-propargyl+metribuzin (54 +120 g/ha) at 30-35 DAS | clodino+Metri | kg | 6.3 | 0.174 |
|  | Water | m3 | 0.17 | 0.5 |
|  | Labour | 15.68 | 0.23 | 8 |
|  | Total |  |  | 3.0212 |
| Control | - | - | - | - |

**Table 10. Total energy equivalent input**

| Treatment details | Fixed energy | N + Residue | Herbicide | Total |
| --- | --- | --- | --- | --- |
| 100% RDN+ Paddy residue +Sulfosulfuron+carfentrazone (25 + 20 g/ha) at 30-35 DAS | 500.345 | 880 | 2.21 | 1382.555 |
| 100% RDN+ Paddy residue + Clodinafop-propargyl +metsulfuron (60 +4 g/ha) at 30-35 DAS | 500.345 | 880 | 2.33 | 1382.675 |
| 100% RDN+ Paddy residue +Clodinafop-propargyl+metribuzin (54 +120 g/ha) at 30-35 DAS | 500.345 | 880 | 3.02 | 1383.365 |
| 100% RDN+ Paddy residue + No herbicide | 500.345 | 880 | 0 | 1380.345 |
| 125% RDN+ Paddy residue +Sulfosulfuron+carfentrazone (25 + 20 g/ha) at 30-35 DAS | 500.345 | 914.34 | 2.21 | 1416.895 |
| 125% RDN+ Paddy residue + Clodinafop-propargyl +metsulfuron (60 +4 g/ha) at 30-35 DAS | 500.345 | 914.34 | 2.33 | 1417.015 |
| 125% RDN+ Paddy residue +Clodinafop-propargyl+metribuzin (54 +120 g/ha) at 30-35 DAS | 500.345 | 914.34 | 3.02 | 1417.705 |
| 125% RDN+ Paddy residue + No herbicide | 500.345 | 914.34 | 0 | 1414.685 |
| 125% RDN+ Paddy residue + waste decomposer +Sulfosulfuron+carfentrazone (25 + 20 g/ha) at 30-35 DAS | 500.345 | 916.265 | 2.21 | 1418.82 |
| 125% RDN+ Paddy residue +waste decomposer+ Clodinafop-propargyl +metsulfuron (60 +4 g/ha) at 30-35 DAS | 500.345 | 916.265 | 2.33 | 1418.94 |
| 125% RDN+ Paddy residue +waste decomposer+Clodinafop-propargyl+metribuzin (54 +120 g/ha) at 30-35 DAS | 500.345 | 916.265 | 3.02 | 1419.63 |
| 125% RDN+ Paddy residue + waste decomposer +No herbicide | 500.345 | 916.265 | 0 | 1416.61 |
| RDN+Sulfosulfuron+carfentrazone (25 + 20 g/ha) at 30-35 DAS | 500.345 | 0 | 2.21 | 502.555 |
| RDN+Clodinafop-propargyl +metsulfuron (60 +4 g/ha) at 30-35 DAS | 500.345 | 0 | 2.33 | 502.675 |
| RDN+Clodinafop-propargyl+metribuzin (54 +120 g/ha) at 30-35 DAS | 500.345 | 0 | 3.02 | 503.365 |
| RDN+ No herbicide | 500.345 | 0 | 0 | 500.345 |

Fig 1. Graphical representation of meteorological data of wheat grown during *Rabi* 2018-19

Fig 2. Graphical representation of meteorological data of wheat grown during *Rabi* 2019-20

**References:**

1. Jackson, M.L. Soil chemical analysis. Asia Publication House, Bombay. pp. 165-167. (1973).
2. Subbiah, B.V. & Asija, G.L.A rapid procedure for estimation of available nitrogen in soil. *Current Science*, **25**: 259-260(1956).
3. Olsen, S.R., Sole, C.V., Watanabe, F.S. & Dean, L.A. Estimation of available phosphorous in soils by extraction with sodium bicarbonate. USDA Circulation, 1939: pp 1-19 (1954).
4. Blake, G.R. & Hartge, K.H. Bulk Density. In: Methods of Soil Analysis. Part 1. Soil Science Society of America Journal, Madison, WI, USA, pp. 363-376 (1986).
5. Choudhary, M., Rana, K.S., Bana, R.S., Ghasal, P.C., Choudhary, G.L., Jakhar, P., Verma, R., Energy budgeting and carbon footprint of pearl millet – Mustard cropping system under conventional and conservation agriculture in rainfed semi-arid agro-ecosystem. *Energy* **9.** 136 (2017).
6. Dagistan, E., Demirats, B, & Yalmaz, Y. Energy usage and benefit- cost analysis of cotton production in turkey. *Afr J Agric. Res .***4**, 599-604 (2009).
7. Mittal, J,P. & Dhawan, K,C. Research manual on energy requirements in agricultural sector. New Delhi: ICAR. p. 150 (1988).
8. Devasenapathy, P., Senthilkumar, G. & Shanmugam, P.M. Energy management in crop production. *Indian J Agron*. **54**, 80-90 (2009).
9. Singh, K.P. Prakash, V., Srinivas, K. & Srivastva, A.K. Effect of tillage management on energy-use efficiency and economics of soybean-based cropping systems under the rainfed conditions in NW Himalayan region. *Soil Res*, **100**, 78e82 (2008).
10. West, T.O., Marland, G., King, A.W., Post, W.M., Jain, A.K. & Andrasko, K. Carbon management response curves: Estimates of temporal soil carbon dynamics. *Environ Manag*. **33**, 507e18 (2004).
11. Lal, B., Gautam, P., Nayak, A.K., Panda, B.B., Bihari, P., Tripathi, R., Shahid, M., Guru, P.K., Chatterjee, D., Kumar, U. & Meena, B.P. Energy and carbon budgeting of tillage for environmentally clean and resilient soil health of rice-maize cropping system. *J Clean Prod*. **226**, 815e30 (2019).
12. Wang, H,, Yang, Y., Zhang, X. & Tian, G. Carbon footprint analysis for mechanization of maize production based on life cycle assessment: a case study in Jilin Province, China. *Sustainability.* **7**, 15772e84 (2015).
13. Deng JL. Grey controlling system. Cent Inst Technol. **10**, 9e18 (1982).
14. Liu, J., Chen, S., Wang, H. & Chen, X. Calculation of carbon footprints for water diversion and desalination projects. *Energy Procedia*. **75**, 2483e94 (2015).
15. Houshyar, E., Dalgaard, T., Tarazkar, M.H. & Jorgensen, U. Energy input for tomato production what economy says, and what is good for the environment. *J Clean Prod.***89**, 99e109(2015).
16. Lal, R. Carbon emissions from farm operations. *Environ Int*. **30**, 981e90 (2004).
